# Pseudomembranous conjunctivitis with hand, foot and mouth disease in a pregnant woman : a case report

**DOI:** 10.1186/s12886-021-01878-7

**Published:** 2021-03-02

**Authors:** Yoo Jin Kim, Tae Gi Kim

**Affiliations:** Department of Ophthalmology, Kyung Hee University Hospital at Gangdong, Kyung Hee University, # 892, Dongnam-ro, Gangdong-gu, 05278 Seoul, Korea

**Keywords:** Hand foot mouth disease, Pseudomembrane, Conjunctivitis, Pregnant

## Abstract

**Background:**

Hand, foot, and mouth disease (HFMD) is a common systemic infection that is caused by an enterovirus, normally Coxsackie A16. Generally, it affects children or immunocompromised adults. Only a few reports have described pseudomembranous conjunctivitis associated with HFMD. We aim to describe the clinical outcomes and ocular findings of a 37-year-old female with HFMD and concurrent severe pseudomembranous conjunctivitis, who was 28 weeks pregnant.

**Case presentation:**

A female patient who was 28-weeks pregnant was referred for an ophthalmological review due to pain and injection in both eyes. The patient was hospitalized under obstetrics and gynecology and evaluated for Behcet’s disease with oral and perineal ulcers. In an ophthalmic examination, both eyes were observed to have a conjunctival injection. Behcet’s disease-associated conjunctivitis was diagnosed. Topical steroids and antibiotics were administered every 6 h. Two days after her presentation, a maculopapular eruption occurred on her palms. Enterovirus type 71 was detected in a serum virus antibody test, and the patient was diagnosed with HFMD. After 7 days, severe pseudomembranous conjunctivitis and corneal epithelial defects occurred in both eyes. Topical steroids were administered every 3 h, and the pseudomembrane was removed every 2 to 3 days. The pseudomembrane did not occur after 3 weeks, but corneal erosion persisted. After 3 months, the corneal erosion had completely resolved.

**Conclusions:**

HFMD-associated conjunctivitis is a rare complication in adults, however it can appear as a severe pseudomembranous conjunctivitis. In this case, the removal of the pseudomembrane and topical steroids helped improve the symptoms.

## Background

Hand, foot, and mouth disease (HFMD) is a common and highly contagious infectious disease caused by an enterovirus, generally Coxsackie A16. However, other serotypes have also been described, such as Enterovirus 71 [1]. HFMD predominantly affects children and immunocompromised adults and is rarely prevalent in immunocompetent adults [1]. It is characterized by mild fever and maculopapular or vesicular eruptions on the extremities and genitalia as well as ulceration of the mouth, palate, and pharynx [2]. Symptoms usually disappear spontaneously within a week. Systemic complications, including respiratory issues, meningoencephalitis, and pericarditis, can also rarely occur.

Although ocular involvement is rare, uveoretinitis, outer retinitis, exudative maculopathy, and occlusive retinal vasculitis have been described [3–10]. However, there are few reports of pseudomembranous conjunctivitis. Brown reports the case of a 9-year-old child with HFMD who had an ulcerated phlyctenule [11]. However, cases of conjunctivitis in adults are rare, and no clinical progress has been reported.

We report the case of a 37-year-old female who was 28 weeks pregnant that was diagnosed with HFMD and concurrent acute pseudomembranous conjunctivitis. Herein, we report the clinical course of this patient, who received topical and systemic treatment.

## Case presentation

A 37-year-old female who was 28 weeks pregnant, was referred to our clinic due to a conjunctival injection and eye pain. The patient was admitted to the obstetrics and gynecology department with a perineal ulcer. Vesicular rashes occurred on her tongue and labial mucosa. She was diagnosed with Behcet’s disease and treated conservatively. One week before the onset of her symptoms, she visited a swimming pool. The patient was healthy, with no history of immunosuppression. On admission, the patient had a body temperature of 36.6 ◦C, a pulse of 78 beats per minute, and blood pressure of 111/63 mmHg.

**Fig. 1:**
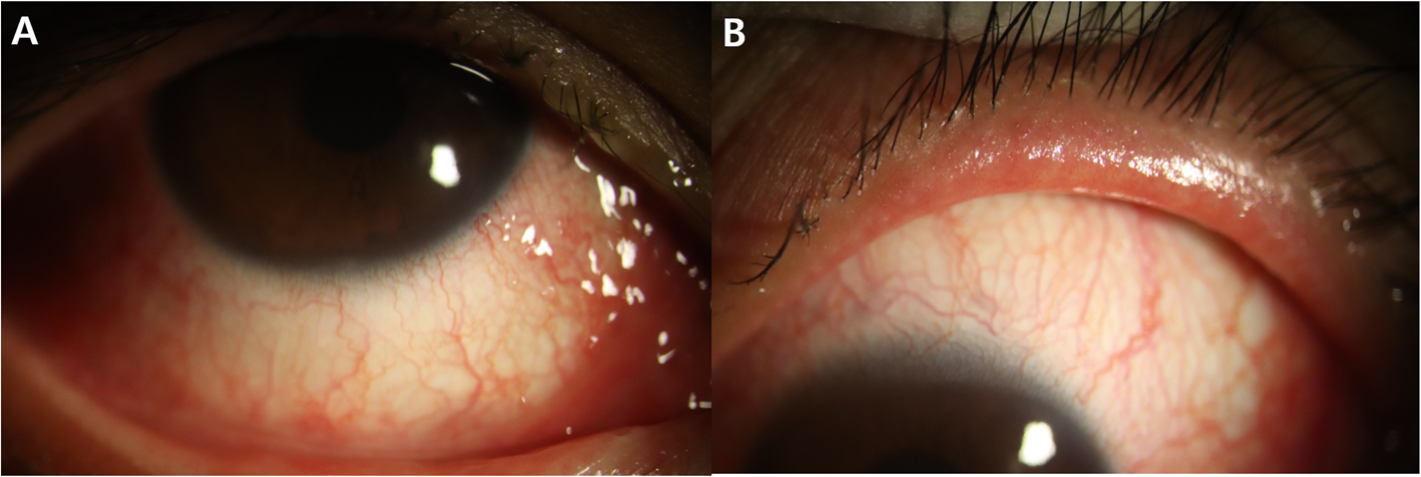
Slit-lamp examination at the initial presentation. Anterior photograph showing a conjunctival injection (**a**) involving the bulbar and palpebral conjunctiva (**b**)

On initial ophthalmological examination, a bilateral bulbar and palpebral conjunctival injection was observed (Fig. 1). A pseudomembrane and follicle were not observed. The patient’s intraocular pressures and anterior segment examination findings were normal in both eyes. Behcet’s disease with ocular involvement was diagnosed. Topical steroids (prednisolone acetate 1 %) and antibiotics (tobramycin 0.3 %) were administered four times a day. Additionally, ophthalmic ointments (Maxitrol, Alcon) were used before bedtime.

**Fig. 2:**
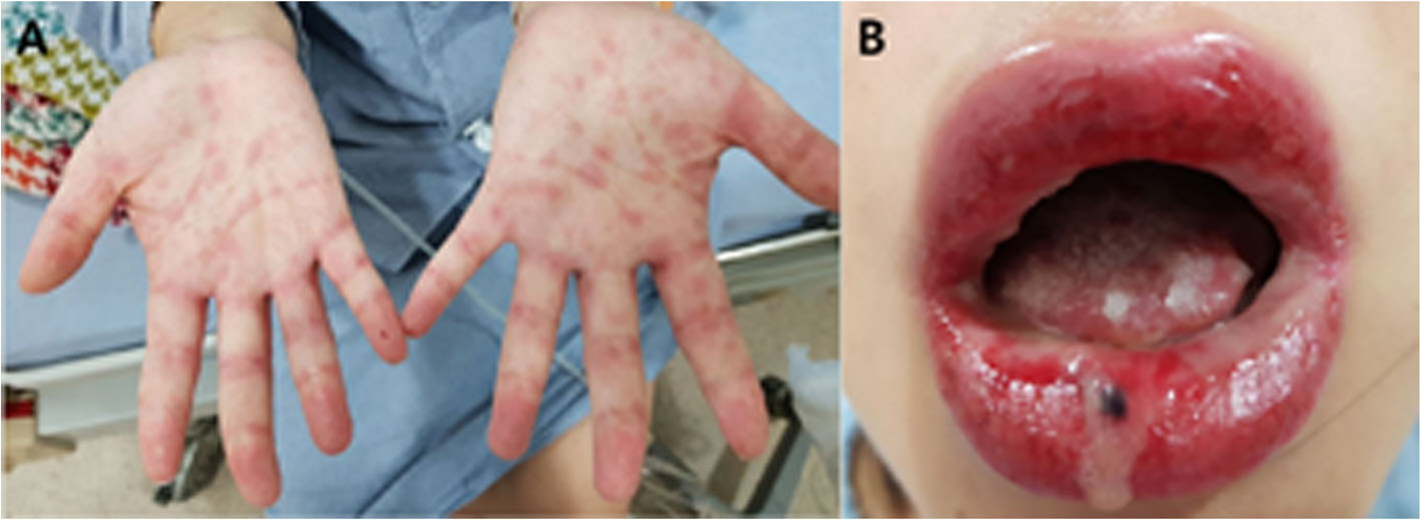
A pruritic eruption of the small vesicles on one palm (**a**) and edematous lips and oral mucosa with erosion and pus (**b**)

Two days later, a maculopapular eruption began to develop on her palms. HFMD was diagnosed based on the clinical findings and distribution being typical of HFMD (Fig. 2). In order to identify the causative virus of HFMD, serum viral titer tests of Coxsackievirus type A16 and Enterovirus type 71 were performed using neutralizing antibody assays. Serum sample analysis was conducted by an external organization (Green Cross Corporation, Youngin, Korea). As per the results, blood test for Enterovirus type 71 antibody titers were positive (titer 1:64, ≥ 1:8) and coxsackievirus A16 antibody titers were negative (titers 1:4, < 1:8). Other viral tests such as Epstein–Barr virus, cytomegalovirus, and human immunodeficiency viruses showed positive results for IgG and negative results for IgM. In addition, the rapid plasma reagin tests displayed negative results. Therefore, we excluded the possibility of other viral and bacterial infections. Furthermore, autoimmune tests such as antinuclear antibody, antineutrophil cytoplasmic antibody, and anti-cyclic citrullinated peptide also showed negative results.

**Fig. 3:**
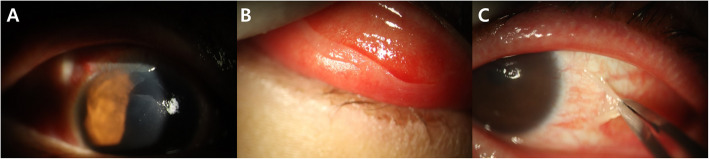
At 7 days, a slit-lamp examination showed a large corneal epithelial defect (**a**) and severe pseudomembrane on the palpebral conjunctiva (**b**) and bulbar conjunctiva (**c**). The pseudomembrane was removed using round-tipped forceps (**c**)

One week after her presentation, a severe pseudomembrane occurred in the bulbar and palpebral conjunctiva, and a defect of the corneal epithelium was observed (Fig. 3a-c). After administering topical anesthetic drops, most of the pseudomembrane in both eyes was removed using a cotton swab and round-tipped forceps (Fig. 3d). Then, topical steroids (prednisolone acetate 1 %) were administered every 3 h. The pseudomembrane was removed every 2 to 3 days for 10 days during a slit-lamp examination.

**Fig. 4:**
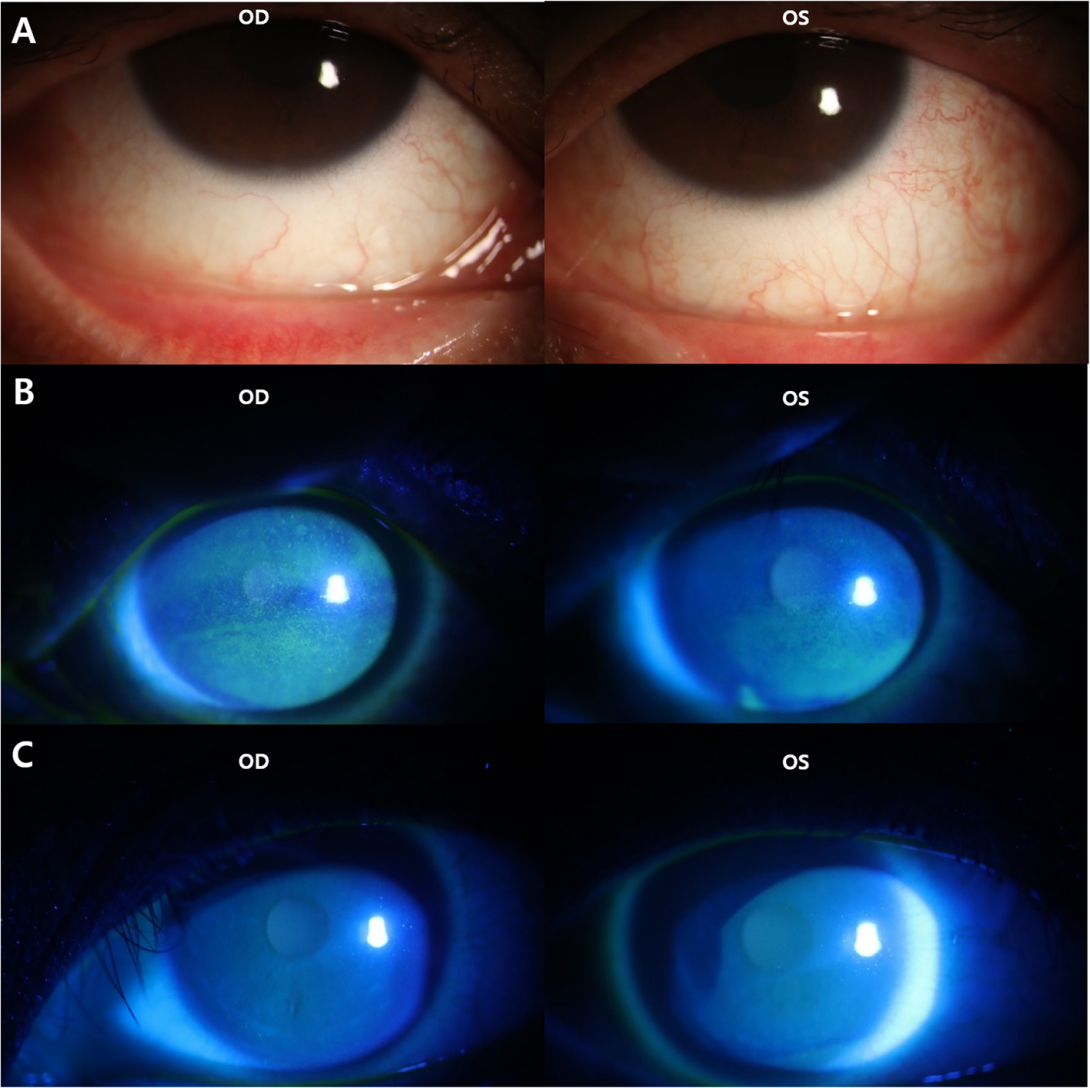
At 3 weeks, no conjunctival pseudomembrane was observed (**a**), but punctate corneal erosion persisted in both eyes (**b**). At 3 months, the punctate corneal erosion in both eyes had completely resolved (**c**)

After 3 weeks, the patient’s conjunctival injection had improved, and a pseudomembrane was not observed. However, punctate corneal erosion was seen in both eyes (Fig. 4). The topical steroids were changed to fluorometholone and were administered every 6 h. Artificial tears and ophthalmic ointment were continuously used. After 2 months, the punctate corneal erosion had improved, and after 3 months, had completely resolved (Fig. 4).

## Discussion and conclusions

HFMD can be associated with conjunctivitis, as reported in a few cases in the literature [11]. Diagnosing HFMD mainly relies on pathognomic clinical findings and can be supported by laboratory tests such as a serum antibody titer or polymerase chain reaction (PCR) [12]. In this case, a PCR was not performed. However, enterovirus type 71 was detected in a serum virus antibody test. Therefore, the patient was diagnosed with HMFD-associated pseudomembranous conjunctivitis due to the characteristic distribution of the lesions [13]. To the best of our knowledge, there are, to date, no reports that describe the clinical course of patients with HFMD-associated pseudomembranous conjunctivitis.

The underlying pathological mechanisms of HFMD-associated pseudomembranous conjunctivitis remain unclear but may be explained by direct viral infection of the conjunctiva through hematogenous spread or due to an autoimmune response. Ocular complications were first described in 1991 by Yannuzzi et al., who focused on unilateral acute idiopathic maculopathy [14]. This condition is associated with Coxsackievirus 16 or an Enterovirus 71 infection, but a number of other serotypes have also been described. Systemic disease caused by Enterovirus 71 is more severe than Coxsackievirus 16 [1]. In the present case, the patient was positive for Enterovirus 71, and therefore was more likely to have more severe ocular complications.

Enterovirus 71 can be found in the saliva, sputum, nasal mucus, and stools. The virus quickly spreads through close contact between individuals, droplets in the air, or by touching contaminated objects. Less commonly, HFMD can be transmitted by swallowing water in a swimming pool that has been contaminated with a stool containing the virus [15–17]. In this case, the patient was likely infected by contaminated water, as the symptoms appeared 7 days after going to a swimming pool.

Herpangina symptoms, including oral ulcers, are caused by viral particles traveling to secondary sites of replication after viremia. Conjunctival inflammation and pseudomembranes can also be estimated as a result of viral replication. Pseudomembranous conjunctivitis is caused by inflammation of the conjunctiva and is characterized by mucopurulent discharge and pseudomembrane formation, which is mainly composed of mucus and fibrin [18, 19]. Management is aimed at reducing ocular inflammation with topical steroids and hydrating the ocular surface with artificial tears and lubricants. Removing the pseudomembrane can help improve symptoms and wound healing. Conjunctival goblet cell loss occurs in ocular surface inflammatory diseases, and this can cause long-term symptoms of dry eye disease [20]. Similarly, in this case, corneal punctate erosion improved after 3 months of treatment. Therefore, when an ocular complication occurs due to HFMD, it is necessary to evaluate and treat long-term dry eye disease.

Specifically, the patient, in this case, was pregnant. In rare cases, HFMD can result in serious infections regardless of pregnancy. According to Giachè et al., 41 % of HFMD-infected pregnant females were symptomatic, and only 15.5 % developed mucosal ulceration, such as oral aphthae [21]. These findings suggest that HFMD in pregnancy is usually asymptomatic or mild. Therefore, it can be assumed that it is very rare that a serious mucocutaneous lesion occurred, as in this case.

An important differential diagnosis of HFMD is erythema multiforme major. Erythema multiforme major is an acute, self-limiting mucocutaneous disease characterized by the abrupt onset of red papules that evolve to target or bull’s eye-like lesions on the dorsal and acral surfaces of the hands and feet as well as the extensor surfaces of the extremities [22]. In this case, the patient was positive for Enterovirus A71. Additionally, the eruption was limited to her palms, and the lesion was not shaped like a target. Therefore, the patient is more likely to have had ocular complications associated with HFMD.

In conclusion, we have described the clinical features of HFMD-associated pseudomembranous conjunctivitis. Although HFMD is most common in infants and children, our case highlights that ocular complications may occur in adults and present with severe pseudomembranous conjunctivitis. Clinicians should be aware of the possibility of HFMD in adult patients with pseudomembranous conjunctivitis.

## Data Availability

Not applicable.

## Abbreviation

HFMD: Hand, foot and mouth disease
